# Th17 Cells and IL-17A in Ischemic Stroke

**DOI:** 10.1007/s12035-023-03723-y

**Published:** 2023-10-26

**Authors:** Jingjing Wang, Yuxiao Gao, Yujia Yuan, Huan Wang, Zhao Wang, Xiangjian Zhang

**Affiliations:** 1 Department of Neurology, Second Hospital of Hebei Medical University, 215 Hepingxi Road, Shijiazhuang, 050000 Hebei China; 2 Hebei Collaborative Innovation Center for Cardio-Cerebrovascular Disease, Shijiazhuang, 050000 Hebei China; 3 Hebei Key Laboratory of Vascular Homeostasis, Shijiazhuang, 050000 Hebei China

**Keywords:** Ischemic stroke, Neuroinflammation, Immunomodulation, Th17 cells, Interleukin-17A

## Abstract

The neurological injury and repair mechanisms after ischemic stroke are complex. The inflammatory response is present throughout stroke onset and functional recovery, in which CD4 + T helper(Th) cells play a non-negligible role. Th17 cells, differentiated from CD4 + Th cells, are regulated by various extracellular signals, transcription factors, RNA, and post-translational modifications. Th17 cells specifically produce interleukin-17A(IL-17A), which has been reported to have pro-inflammatory effects in many studies. Recently, experimental researches showed that Th17 cells and IL-17A play an important role in promoting stroke pathogenesis (atherosclerosis), inducing secondary damage after stroke, and regulating post-stroke repair. This makes Th17 and IL-17A a possible target for the treatment of stroke. In this paper, we review the mechanism of action of Th17 cells and IL-17A in ischemic stroke and the progress of research on targeted therapy.

## Introduction

Ischemic stroke (IS) is one of the leading causes of death and disability worldwide. It is increasing in prevalence with increasing aging and the growing prevalence of diabetes, hypertension, and obesity. The blocked blood flow leads to a series of cascade responses that gradually expand from the ischemic core to the peripheral penumbra, including excitotoxicity, calcium overload, mitochondrial damage, oxidative stress, apoptosis, autophagy, neuroinflammation, and blood–brain barrier damage [1, 2]. Among them, the inflammatory response is a significant link in a highly complex cascade of reactions throughout stroke onset and repair. A growing body of evidence suggests that intense neuroinflammation is the primary mechanism of secondary brain injury in the early stages of stroke. In contrast, in the later stages, the inflammatory response promotes neurogenesis, angiogenesis, and neuronal plasticity, thereby facilitating neurofunctional recovery [3].

**Fig. 1:**
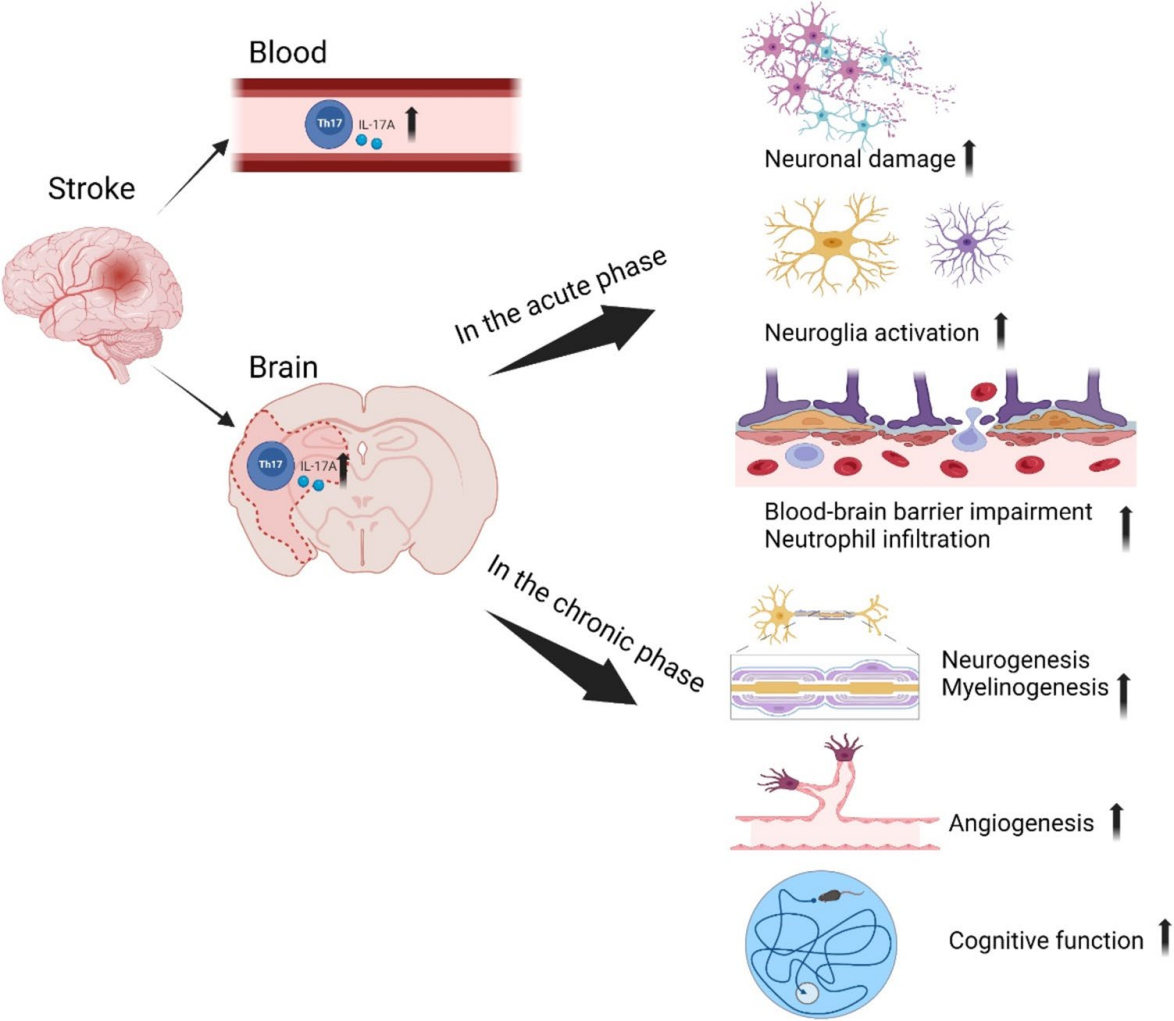
Graphical abstract of this review. Stroke causes increased levels of Th17 cells and IL-17A in peripheral blood and brain tissue. Th17 and IL-17A play different roles in the acute and chronic phases of stroke. In the acute phase, Th17 and IL-17A cause secondary brain damage by directly damaging neurons, promoting glial cell activation, disrupting the blood–brain barrier, and promoting peripheral immune cell infiltration. However, in the chronic phase, Th17 and IL-17A promote neurogenesis, myelinogenesis, angiogenesis, and cognitive improvement

T cells play an important role in post-stroke neuroinflammation. Th17 cells are a subset of CD4 + T cells, and their most important cytokine is IL-17A [4]. In recent years, with the advancement of research, it has been gradually recognized that Th17 and IL-17A play a key role in the pathogenesis of inflammation and autoimmune diseases [5–7]. A growing number of studies have shown that IL-17A acts on multiple resident cells in the central nervous system, enhancing the neuroinflammatory response after stroke and exacerbating ischemic brain injury. We review the function of Th17/IL-17A, the mechanism of action of Th17/IL-17A in stroke, and Th17/IL-17A-related stroke therapy (Fig. 1).

## Differentiation of Th17 Cells and IL-17A Signaling Pathway

CD4 + Th cells are involved in the clearance of pathogens as a key factor of adaptive immunity. Naive CD4 + T cells mature in the thymus and enter the peripheral lymphoid organs for circulation, including the spleen, lymph nodes, and mucosa-associated lymphoid tissue (MALT). Naive CD4 + T cells receive antigen from antigen-presenting cells (APCs) and rapidly activate, proliferate, and differentiate into different Th lineages, including Th1, Th2, Th17, and T regulatory (Treg) [8]. The fate of Th lineages depends not only on the type and activation status of APCs but also on the type of pathogen-associated molecular pattern (PAMP) or damage-associated molecular pattern (DAMP), the strength of T cell receptor (TCR) signaling, the control of environmental signals (mainly cytokines), and the induction of specific transcription factors (TFs) within T cells. In 2006, researchers found that naive T cells differentiate into a new type of CD4 + Th cells, Th17 cells, in the combined effect of transforming growth factor-β (TGF-β) and interleukin-6 (IL-6) [4]. Th17 cells characteristically express retinoic acid-related orphan receptor γt (RORγt) and secrete a unique set of pro-inflammatory cytokines (IL-17A, IL-17F, IL-21, IL-23, IL-6, Interferon-gamma(IFN-γ), and granulocyte–macrophage colony-stimulating factor(GM-CSF)) [9]. Th17 cells are present in small numbers in circulation and large numbers in mucosal tissues [10].

**Fig. 2:**
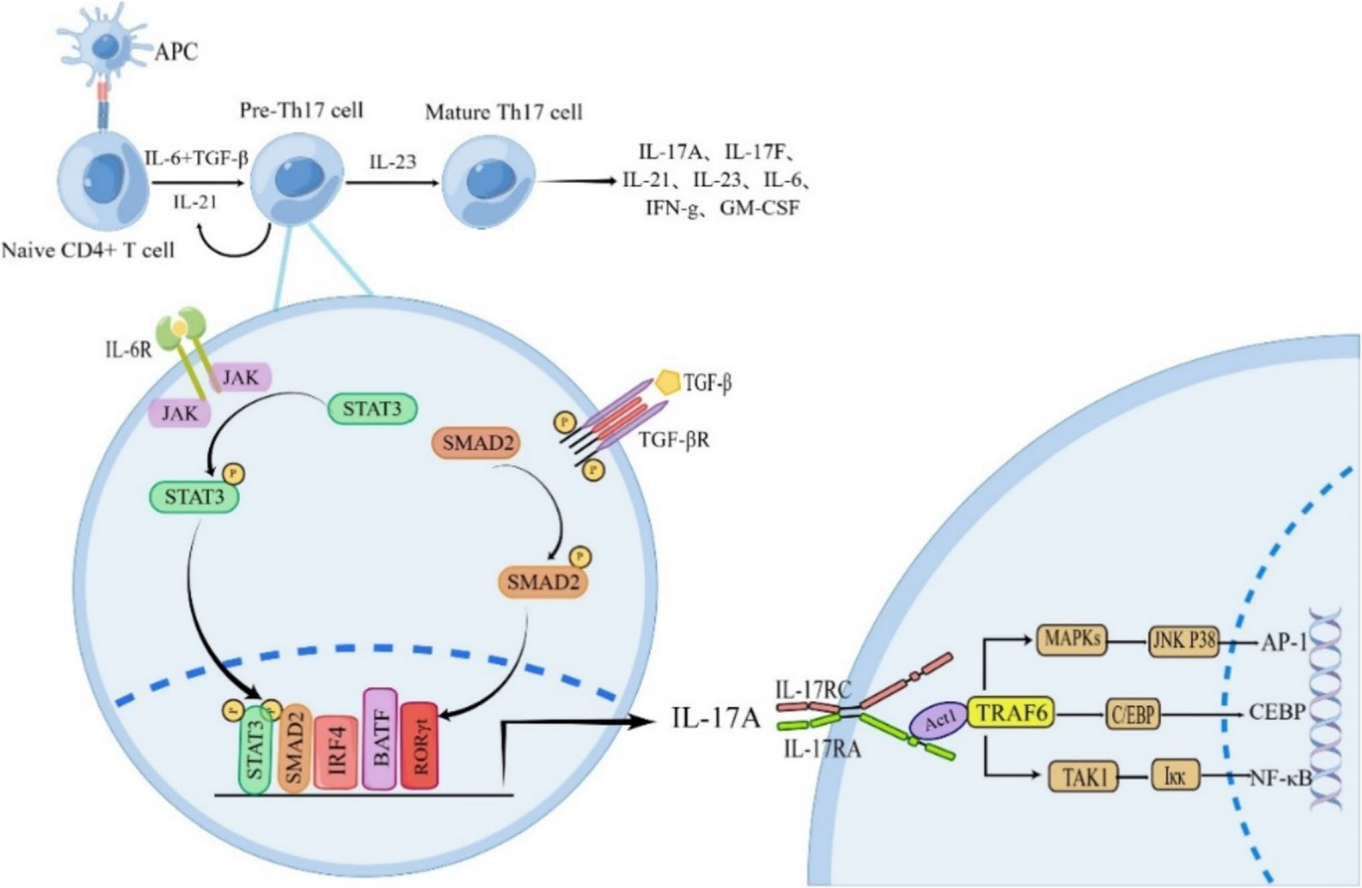
Differentiation of Th17 cells and IL-17A signaling pathway. Th17 cells are differentiated from naive CD4 + T cells. CD4 + T cells receive stimulation from APCs and are affected by IL-6 and TGF-β to initiate the initial differentiation program. IL-21 is produced by Th17 cells in an autocrine manner and then promotes the proliferation and differentiation of Th17 cells during the expansion phase. IL-6 binding to IL-6R mediates the phosphorylation of STAT3 by JAK2. TGF-β binding to its receptor mediates the phosphorylation of SMAD2. After nuclear translocation, P-STAT3, P-SMAD2, BATF, IRF4, and RORγt bind to the IL- 17 promoters to initiate IL-17A transcription. After IL-17 binds to its receptor, the intracellular structure of this receptor recruits and activates Act1. Activated Act1 phosphorylates TRAF6 and triggers TRAF6-dependent transcription of target genes such as NF-κB, CEBP, and MAPK/AP-1

The differentiation and maturation of Th17 cells are divided into three stages (Fig. 2) [11]. In the initial phase, the combination of TGF-β and IL-6 or IL-21 triggers the initial differentiation of naive CD4 + T cells into precursor Th17 cells. It was shown that IL-6 plays a dominant role in the initial stage of differentiation [12], while IL-21, at this stage, acts only as an alternative pathway to IL-6. However, during the second phase of expansion, IL-21, produced by Th17 cells, plays a significant role in the autocrine expansion cycle, ultimately promoting the differentiation and proliferation of Th17 cells and the production of the IL-23 receptor (IL-23R) [13]. During the final maturation phase of Th17 cells, IL-23 binds to IL-23R to achieve complete and sustained differentiation of Th17 cells and promote the secretion of the pro-inflammatory factor IL-17A in large quantities [8]. Although the mechanisms of differentiation are complex, several key pathways activated by cytokines be involved in the development of Th17 cells (Fig. 2) [14]. The binding of IL-6 to its receptor IL-6R leads to the activation of the janus kinase 2 (JAK2)/signal transducer and activator of transcription 3(STAT3) signaling pathway [12]. Activated STAT3 enters the nucleus and activates the expression of transcription factors RORγt and RORα. TGF-β binds to its receptor and mediates the nuclear translocation of Recombinant Mothers Against Decapentaplegic Homolog 2 (SMAD2), which initiates the transcription of IL-17A [9]. Two other transcription factors, B-cell activating transcription factor (BATF) and interferon regulatory factor 4 (IRF4), which are independent of STAT3 and SMAD2 signaling, are also essential for Th17 differentiation [15]. STAT3, RORγt, SMAD2, BATF, and IRF4 form a complex that together binds the promoter of the IL-17A gene to promote Th17 differentiation and IL-17A expression. However, it is not clear how BATF and IRF4 crosstalk with STAT3 to regulate Th17 differentiation.

IL-17A (also known as IL-17), a marker cytokine of the Th17 cell subpopulation, was discovered in 1993 and became the founding member of the IL-17 family. In addition to being primarily derived from Th17 cells, IL-17A is also expressed in CD8 + cells, γδ T cells, natural killer T (NKT) cells, group 3 innate lymphoid cells (ILC3s), neutrophils, and microglia [16]. The remaining five members of the IL-17 family include IL-17B, IL-17C, IL-17D, IL-17E, and IL-17F. Studies have shown the highest homology between the amino acid sequences of IL-17A and IL-17F. Moreover, heterodimers of both exist in vivo (IL-17A/F) [17]. IL-17A, IL-17F, and IL-17A/F share the same receptor (IL-17RA/RC complex) [18] and exhibit similar pro-inflammatory effects in many diseases. IL-17 receptors are widely expressed in the central nervous system, such as neurons, glial cells, and brain endothelial cells. IL-17A binds to the IL-17RA/RC complex and recruits the ubiquitin ligase Act1 through the intracellular structure of this receptor (SEFIR structural domain). Act1 recruits tumor necrosis factor (TNF) receptor-associated factor 6 (TRAF6), leading to the activation of nuclear factor kappa B (NF-kB), C/EBP, and the mitogen-activated protein kinase (MAPK)/AP-1 pathways [19, 20].

## Changes of Th17/IL-17A in Ischemic Stroke

Several studies have shown that Th17 cells and IL-17A are present in high numbers in the peripheral blood of patients with IS and are positively associated with severity, poor prognosis, and stroke sequelae such as cognitive impairment. The amount of Th17 cells in the peripheral blood of healthy adults is minimal, accounting for about 1%-4% of CD4 + T cells. Li et al. study suggested that the levels of Th17 cells and IL-17A in the peripheral blood of healthy adults are age-dependent [21]. The percentage of Th17 cells was 2.31%, 1.42%, and 0.94% in healthy elderly, middle-aged, and young adults. IL-17A levels were 27.17, 18.92, and 15.98 pg/ml in the three groups. A study showed that the Th17 cell percentage of patients with IS was approximately 5.8% on admission and was positively correlated with NIHSS scores. The Th17 cell percentage increased to 6.8% on the first day after admission (D1), increased to 7.7% on the third day (D3), and decreased to 5.8% on the seventh day (D7) [22]. Another study suggested a similar trend of Th17 cell changes [23]. On the D1 and D5 after IS, the Th17 percentage was 4.68% and 3.65% and decreased to 2.49% on the D10, similar to healthy controls. The study also documented an increase in IL-17A from 118 pg/ml to 171, 147, and 146 pg/ml on the D1, D5, and D10 compared to controls. Lu et al. recorded a baseline (at admission) Th17 percentage of 4.1% and baseline IL-17 levels of 98.7 pg/ml in IS patients [24]. In addition, in patients’ postmortem stroke tissues, positive staining of cells expressing IL-17A was higher in the infarcted area than in non-ischemic tissues and remained at higher levels on days 2–5 [25].

Numerous studies have suggested that in animal ischemic stroke models, Th17 cell and IL-17A levels in the brain and circulation tend to increase after infarction. IL-17A in brain tissue was enhanced within one day, peaked on day 3, and decreased slightly in the following days, and this trend has been confirmed by several experimental results [20, 26, 27]. Luo’s study showed that the percentage of Th17 cells in brain tissue increased from less than 5 to 30% at 6 h after transient middle cerebral artery occlusion (tMCAO) and to 50% at 72 h after tMCAO [28]. However, Guo’s study showed that the percentage of Th17 cells rose to approximately 10% at 72 h after tMCAO [29]. This discrepancy may be because Luo’s study defined IL-17A-releasing T cells as Th17 cells, which led to an expansion of the Th17 cell range. Nevertheless, the trend of elevated Th17 cells in brain tissue after MCAO is undeniable.

## Mechanism of Action of Th17 Cells and IL-17A in Ischemic Stroke

### Th17 Cells and IL-17A Promote the Pathogenesis of Ischemic Stroke

Thrombosis or thromboembolism due to atherosclerosis (AS) is the main pathogenesis of stroke. When vascular endothelial dysfunction occurs, low-density lipoprotein (LDL) particles penetrate the intimal layer and activate macrophage-derived foam cells to secrete pro-inflammatory factors, leading to plaque growth and lipid nucleation [30]. On the other hand, immune inflammation caused by immune cell infiltration, especially T cells, is also a key mechanism that promotes AS. In recent years, an increasing number of studies have shown that Th17 and IL-17A promote the onset and development of atherosclerosis. Liu et al. suggested that Th17 cells were positively associated with carotid atherosclerotic plaques and that peripheral blood Th17 cell levels were higher in patients with unstable plaques [31]. IL-17A promotes thrombosis and callogenesis by activating tissue factors and reducing anticoagulation mediators (CD39 and thrombomodulin) [32]. Both in situ thrombosis and embolism due to unstable plaque detachment are important pathogenic mechanisms of IS. Animal studies have shown that atherosclerotic plaques in ApoE-/- mice contain higher levels of Th17 cells and IL-17A than in wild-type (WT) mice, and exogenous supplementation with IL-17A significantly increases plaque size. All of the above studies suggest that Th17 cells and IL-17A are associated with AS [33, 34]. Studies on the mechanisms promoting AS progression have shown that IL-17 may promote migration and adhesion of innate immune cells (neutrophils, monocytes, and macrophages) to vascular lesions [34] and promote the release of pro-inflammatory mediators [35] by acting on the three cell layers of the vessel wall. In vitro studies have shown that IL-17A induces the secretion of pro-inflammatory cytokines (IL-6, GM-CSF) and chemokines (IL-8, C-X-C motif chemokine 1(CXCL1)) from human vascular endothelial cells (HVECs) through activation of STAT3 phosphorylation and nuclear translocation, and further induces neutrophil recruitment [36, 37]. In addition, IL-17A can also induce the expression of adhesion molecules in HVECs to promote the adhesion and rolling of monocytes and platelets to endothelial cells [35, 38]. IL-17A can promote the expression of Vascular cell adhesion molecule 1(VCAM-1) in vascular smooth muscle cells (VSMCs) by a mechanism that may be related to the MAPK/ERK/NF-κB signaling pathway [39]. In addition, IL-17A induces significant expression of several other chemokines in VSMCs, such as CC chemokine ligand(CCL)20, CCL5, monocyte chemotactic protein-1 (MCP-1), CXCL16, and the cytokine IL-6, promoting leukocyte recruitment and vascular inflammation [40]. When IL-17A is co-cultured with human atherosclerotic plaques, IL-17A induces the release of more MCP-1 and matrix metalloproteinase 9 (MMP-9) mRNA as well as IL-6, TNF-α, G-CSF, and TGF-β proteins [35]. New studies have found that IL-17 induces senescence in endothelial cells [21], which is one of the main causes of structural changes and dysfunction in blood vessels and is the basis of AS. However, it has also been shown that IL-17A deficiency can lead to the formation of atherosclerotic plaques, suggesting a protective role for IL-17A [41]. The promotive or inhibitory effect of IL-17A on AS is an important target for preventing cardio-cerebrovascular diseases and needs further exploration.

### Th17 Cells and IL-17A Promote Secondary Brain Injury After Ischemic Stroke

After IS, IL-17 is released by a variety of central and peripheral immune cells. When cerebral ischemia occurs, injured cells release DAMPs and first activate the innate immune response. As early as 12 h after ischemia/reperfusion (I/R), microglia increase the levels of pro-inflammatory cytokines, including IL-17A, IL-23, IL-β, and TNF-α, by upregulating and activating Toll-like receptors 2(TLR2) and sphingosine kinase 1(Sphk1) signaling molecules [42]. During this period, astrocytes also act as an important source of IL-17A release, increasing IL-17A levels in ischemic brain tissue and cerebrospinal fluid [43]. IL-23 released from astrocytes can stimulate microglia to produce more IL-17 and other inflammatory mediators such as IL-6, macrophage inflammatory protein-2 (MIP-2), and inducible nitric oxide synthase (iNOS) [44]. Infiltrating intrinsic immune cells, γδ T cells, are the primary source of IL-17A for 12 h to 3 days after stroke [45, 46]. The adaptive immune response is activated after the innate immune response. CD4 + T cells increase in brain tissue 24 h after tMCAO, with their peak occurring six days later and continuing until at least 30 days [47]. Infiltrating CD4 + T cells driven by APCs and specific cytokines (IL-6/IL-21 + TGF-β) differentiate into Th17 cells.IL-23, secreted by macrophages, microglia, and astrocytes, stabilizes the structure of Th17 cells and is an important factor in limiting the production of IL-17 by Th17 cells [45]. Ischemic signals (DAMPs) transmitted to the periphery activate RAGE receptors on naive CD4 + T cells and promote the differentiation of CD4 + T cells to Th17 cells after MCAO by directing the reprogramming of fatty acid metabolism [48]. Th17 cells in the peripheral circulation also peak 3–5 days after ischemia, secreting pro-inflammatory factors such as IL-17A and crossing the disrupted blood–brain barrier(BBB) to reach the ischemic zone.

#### Th17 Cells and IL-17A Enhance BBB Damage After Ischemic Stroke

**Fig. 3:**
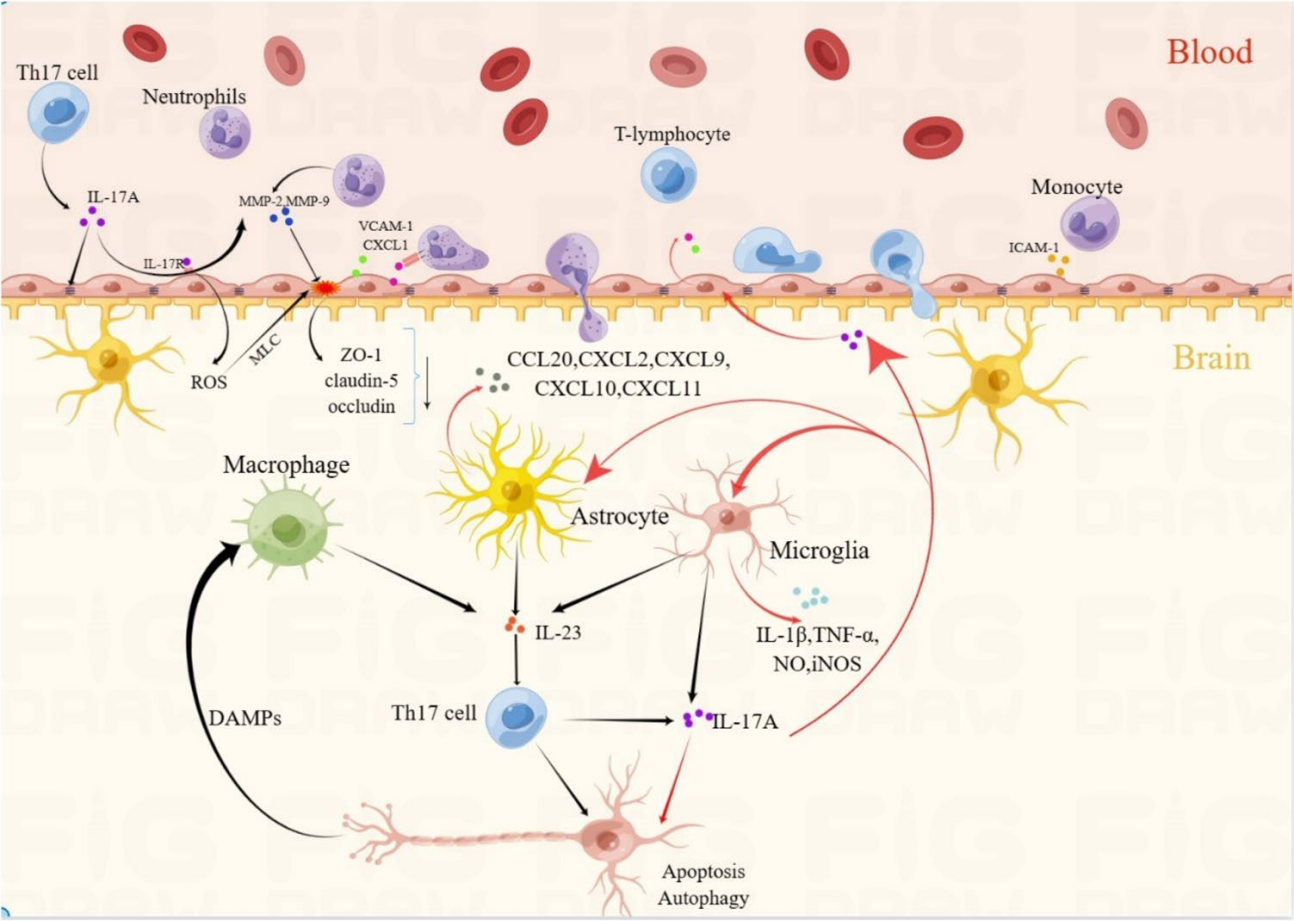
IL-17A-mediated immune response after stroke. IL-17 is mainly produced by peripheral Th17 cells, γδ T cells infiltrating the CNS, and astrocytes and microglia in the brain parenchyma. IL-17A acts on a variety of cells in the CNS and exerts a role in exacerbating brain injury. IL-17A binds to IL-17A receptors on vascular endothelial cells and induces destruction of the BBB by decreasing the endothelial intercellular junction proteins ZO-1, claudin-5, and occludin. IL-17A activates ROS release from vascular endothelial cells and further activates MLC, leading to endothelial cell contraction and gap expansion. The compromised blood–brain barrier creates prerequisites for the influx of peripheral immune cells into the brain parenchyma. IL-17A stimulates the release of chemokines and adhesion molecules from vascular endothelial cells and astrocytes, attracting neutrophils, lymphocytes, and monocytes for adhesion and transendothelial transport. In the brain parenchyma, IL-17A stimulates astrocytes and microglia to produce large amounts of pro-inflammatory mediators. In addition, IL-17 can act alone or synergistically with other factors to directly cause apoptosis and excessive autophagy in neurons

After the ischemic stroke, BBB dysfunction begins with vascular endothelial cell injury due to ischemia. Then, immune cells and molecules act directly or indirectly on BBB components, exacerbating the disruption of BBB structure and increasing permeability, further leading to edema and inflammatory responses in the ischemic area [49]. Th17 cells and IL-17A are involved in BBB destruction in multiple sclerosis (MS) and autoimmune encephalomyelitis (EAE) disease models [50], and it is reasonable to suspect that IL-17-producing cells also contribute to BBB dysfunction after stroke (Fig. 3). IL-17 receptors widely expressed in vascular endothelial cells, neurons, and glial cells are elevated after MCAO [51]. In an in vitro BBB model in which endothelial cells are co-cultured with astrocytes, IL-17A decreases the connexin ZO-1. It synergizes with IL-6 and TNF-α to decrease the expression of claudin-5 and occludin and increase the permeability of the BBB [52]. Zhang’s study showed that IL-17A induces BBB destruction by reducing the expression levels of occludin and claudin-5 proteins and increasing the expression levels of MMP-2 and MMP-9 proteins in vascular endothelial cells [53]. In addition, IL-17A activates IL-17A receptors on vascular endothelial cells, promoting the release of reactive oxygen species (ROS) and further activating the myosin light chain (MLC). Phosphorylated MLC interacts with cytoskeletal actin to induce brain microvascular endothelial cells (BMECs) contraction, ultimately leading to the widening gap between endothelial cells and increased BBB permeability [54]. It has also been shown that IL-17 induces apoptosis in endothelial cells by activating caspase-3 and caspase-9 and upregulating the Bcl2 Associated X Protein(Bax)/ B-cell lymphoma-2 (Bcl-2) ratio, thereby mediating the destruction of BBB [55]。

#### Th17 Cells and IL-17A Promote Infiltration of Peripheral Immune Cells After Ischemic Stroke

IL-17 promotes the adhesion and transendothelial transfer of neutrophils, lymphocytes, and monocytes after stroke (Fig. 3). After recruitment from the periphery, neutrophils adhere to the brain endothelium around the infarct site within minutes and peak 2–3 days after the onset of ischemia [56]. Neutrophils promote the degradation of endothelial cells and BBB by activating MMP-2 and MMP-9 [57]. Activated neutrophils entering the brain parenchyma through the damaged BBB can lead to neuronal death [56]. Lymphocyte infiltration occurs relatively late. In the tMCAO model, peak T-cell infiltration occurs 3–5 days after stroke induction. In the permanent MCAO (pMCAO) model, peak T-cell infiltration occurs at a relatively delayed 7 days after stroke [47]. IL-17A stimulates brain endothelial cells to express VCAM-1 and induces the release of chemokines CCL2 and CXCL1 in a dose-dependent manner, thereby driving neutrophils and T lymphocytes (including Th17 cells) into the brain parenchyma and propagating immune responses [58]. Early brain-derived IL-17 from γδ T cells has also been shown to induce neutrophil infiltration of brain parenchyma by stimulating astrocytes to secrete CXCL1 [59]. Inhibition of the IL-17A signaling pathway (anti-IL-17A treatment) significantly inhibits neutrophil infiltration and reduces infarct size. IL-17A derived from Th17 cells interacts with IL-17 receptors on endothelial cells to induce BBB breakdown by disrupting tight junctions, ultimately leading to massive infiltration of CD4 + T lymphocytes into the central nervous system (CNS) parenchyma [50]. In addition, IL-17 has been shown to support monocyte migration in the blood–brain barrier through an intracellular adhesion molecule (ICAM)-1-dependent mechanism [54].

#### Interaction of Th17 Cells and IL-17A with Glial Cells After Ischemic Stroke

It was found that Th17 cells crosstalk with microglia after stroke (Fig. 3). Th17 and IL-17A can promote microglia differentiation to M1 phenotype, inhibit M2 polarization, and promote the inflammatory response. Meanwhile, M1 microglia secrete IL-6 and IL-23 and recruit and induce differentiation of Th17 cells. Zhao et al. showed that non-invasive vagus nerve stimulation (nVNS) attenuated ischemia–reperfusion injury in mice by promoting microglia M2 polarization. The researchers further nullified the nVNS-induced facilitation of microglia M2 polarization by intranasal injection of recombinant IL-17A, thus demonstrating that IL-17A inhibits the process of microglia M2 polarization after I/R [60]. In I/R model mice, IL-17A knockout (IL-17A^−/−^) or anti-IL-17A monoclonal antibody treatment significantly reduces microglial activation and induces a shift in activated microglia from M1 to M2 phenotype [61]. In OGD-induced microglia, ROS, HMGB1, and IL-17A are expressed increased. Moreover, the upregulation of IL-17A expression mediates the expression of a series of factors and affects p53 and PI3K/Akt signaling pathways, inhibiting microglia proliferation and promoting apoptosis [62]. Microglia recognize I/R-induced DAMPs signaling through TLR receptors, leading to the release of pro-inflammatory cytokines IL-1β, TNF-α, IL-17, and IL-23 [42]. Th17 and γδ T cells express many IL-23R on their surface. IL-23R binds to IL-23 to promote the conversion of Th17 and γδ T cells to a mature neurotoxic phenotype and the release of inflammatory cytokines such as IL-17A [63]. Thus, a positive feedback loop is formed between microglia and Th17, and Th17 becomes a pro-inflammatory factor in brain injury. However, in vitro studies have shown that Th1-related factors can directly activate and trigger pro-inflammatory M1-type gene expression profiles in microglia, whereas Th17 cells or their related factors have little effect on microglia [64].

Astrocytes are also important responders to IL-17A signaling. IL-17A attenuates apoptosis in primary cultured cortical astrocytes by inhibiting OGD/R-induced downregulation of IL-17A receptor membrane translocation [65]. IL-17A antibody significantly inhibits astrocyte activation in the peri-infarct region 3 and 7 days after tMCAO. Stimulation of astrocytes in vitro by TNF-α and IL-17A increases the expression of several chemokines, including CCL20, CXCL2, CXCL9, CXCL10, and CXCL11 [66]. Additional studies have shown that the combination of IL-6 with IL-17A modulates CCL20 expression in astrocytes and increases the migration of CCR6-expressing T cells, including Th17 cells, whereas IL-17A alone has little effect [67]. Astrocytes may also be a source of IL-23 during cerebral ischemia and are involved in the differentiation of Th17 cells as APCs [68]. Astrocytes also secrete IL-17A and are the main cellular source of IL-17A 28 days after tMCAO [69].

#### Th17 Cells and IL-17A Promote Neuronal Injury

In vitro studies show that IL-17A binds to upregulated IL-17A receptors and promotes hippocampal neuronal damage in response to OGD stress [51]. Neuronal apoptosis is one of the major pathological changes of ischemic brain injury. Studies suggest that endoplasmic reticulum stress (ERS) is a key factor in inducing neuronal apoptosis. Using bioinformatics analysis, Zhang et al. found that in blood specimens from stroke patients, ERS-related genes were mainly enriched in immune-related pathways, especially neutrophil activation, and Th17 cell differentiation, and ERS-related proteins specifically included the hypoxia-inducible factor family and cAMP-response element-binding protein (CREB) family [70]. Transient receptor potential cation channel 6 (TRPC6) phosphorylates CREB to activate brain-derived neurotrophic factor (BDNF) and anti-apoptotic protein Bcl-2. TRPC6/CREB pathway maintains neuronal survival and function after stroke by enhancing hypoxia tolerance of neuronal cells [71]. IL-17A may promote I/R-induced neuronal death and neurological dysfunction by increasing calpain-mediated TRPC6 protein hydrolysis [72]. In addition, IL-17A can cause excessive neuronal autophagy to aggravate ischemic injury in tMCAO mice. The Calcineurin/Protein Phosphatase (PP)2B is a Ca2 + -associated Ser/Thr phosphatase and has been proven physically binding to Mammalian Target of Rapamycin(mTOR) [73]. In OGD/R-induced cortical neurons, IL-17A increases PP2B activity and PP2B-mediated dephosphorylation of mTOR to induce excessive autophagy in neurons [74].

### Th17 Cells and IL-17A Regulate Ischemic Stroke Recovery

#### Th17 Cells and IL-17A and Neurovascular Remodeling During Stroke Recovery

During recovery from ischemic stroke, neurogenesis that occurs mainly in the subgranular zone (SGZ) of the dentate gyrus (DG) in the hippocampus and the subventricular zone (SVZ) on the outer walls of the lateral ventricles makes a prominent contribution to the recovery of neurological function. Neural stem cells (NSCs) undergo proliferation and division and differentiate into neural progenitor cells (NPCs), which migrate to the lesion area and differentiate into newborn neurons. The role of IL-17A in regulating neurogenesis remains controversial. In vitro studies have shown that IL-17A inhibits the proliferation of NSCs and the differentiation of NSCs to astrocytes and oligodendrocyte precursor cells (OPCs) [75]. Acute rapid eye movement (REM) sleep deprivation inhibits adult hippocampal neural progenitor cell proliferation by increasing IL-17A expression and activating the p38 MAPK signaling pathway [76]. IL-17A knockout mice show more mature and immature neurons in the hippocampal dentate gyrus and stronger short-lived presynaptic plasticity [77]. IL-17 knockout mice increase the expression of PI3K/Akt pathway-related genes and promote NSC proliferation and neurogenesis from 3 to 28 days after stroke [78]. Another study showed that IL-17 knockout mice upregulated the Wnt signaling pathway after stroke, promoting neurogenesis in the hippocampus and improving cognitive dysfunction after stroke. In vitro experiments also demonstrated that IL-17A downregulated the expression of Wnt2, β-catenin, and GSK-3β and significantly inhibited the growth and proliferation of neurospheres in NSCs [79]. In contrast, Lin and Zhang showed that after tMCAO, activated astrocytes increased IL-17 secretion and improved proliferation and differentiation of NPCs after stroke by regulating the p38 MAPK/calpain 1 signaling pathway and NF-κB factors [69, 80]. IL-17 induces neurite outgrowth in post-sympathetic ganglion neurons by activating NF-κB signaling and inhibiting voltage-dependent Ca2 + influx. The mechanism of IL-17A action on neurogenesis is undoubtedly complex. One study proposed that IL-17A regulates adult neurogenesis in two stages. They suggest that IL-17A may reduce the proliferation and self-renewal of NPCs, which means that it inhibits the early stages of neurogenesis. However, IL-17A promotes the differentiation and maturation of NPCs by downregulating Notch signaling and upregulating FGF-13 expression, which means it increases the later stage of neurogenesis [81].

Oligodendrocytes are the main myelin producers and are susceptible to damage after IS. During ischemic stroke pathology, endogenous oligodendrocytes are induced to be produced. Oligodendrocyte precursor cells (OPCs) are recruited to the demyelinated region and differentiate into mature oligodendrocytes. OPCs transplantation promotes motor and cognitive function recovery in tMCAO mice after 5 weeks. This benefit can be attributed to the enhanced endogenous oligodendrocyte production and promotion of neurite growth and synaptogenesis [82]. It was shown that after exposure to IL-17A for 48 h, OPCs showed increased expression of voltage-gated K + (Kv)1.3 channel protein and decreased expression of phosphorylated Akt (p-Akt), and OPC proliferation was delayed. Blocking Kv1.3 increased p-Akt and prevented IL-17-induced loss of cell viability and proliferation inhibition. These together suggest that IL-17A attenuates Akt signaling through the Kv1.3 channel and inhibits the proliferation and differentiation of OPCs [83]. Wang et al. showed that in a co-culture system of OPCs and astrocytes, IL-17-induced activation of NOTCH1 in OPCs induced the formation of a complex between the adaptor protein Act1 and the NOTHC1 intracellular domain (NICD1). The Act1-NICD complex was translocated into the nucleus to induce inflammatory gene expression and also through some target genes (e.g., STEAP4), promoting OPC proliferation but interfering with OPC maturation [84].

Angiogenesis is an important protective mechanism to promote neurogenesis, neuronal plasticity, and finally functional recovery during after stroke. Different studies have shown that angiogenesis occurs after cerebral ischemia in different steps, such as endothelial cell proliferation and migration, angiogenic sprouting, lumen formation, and endothelial network maturation [85]. The ischemic penumbra secretes numerous angiogenic factors, including vascular endothelial growth factor (VEGF), angiopoietins, platelet-derived growth factor (PDGF), angiogenin, transforming growth factors (TGFs), basic fibroblast growth factor (bFGF), MMP, NO, etc., of which the most important stimulatory factor is VEGF [85]. IL-17 knockout (IL-17 KO) mice show lower expression of VEGF and CD34 than wild-type mice at 28 days of reperfusion injury [86]. There are evidences showing that enriched environment (EE) can mediate angiogenesis in reperfusion-injured rats by increasing the expression of IL-17A in astrocytes. EE increases the level of microvessel density (MVD) in the penumbra and promotes the expression of CD34, VEGF, IL-6, JAK2, and STAT3 [87]. Although there are fewer studies on IL-17A and angiogenesis after stroke, the role of IL-17A on angiogenesis in diabetes mellitus [88] and inflammatory diseases such as arthritis [89, 90] and allergic pulmonary [91] has been demonstrated. It is reasonable to believe that IL-17A will be an important target for post-stroke angiogenesis.

#### Th17 Cells and IL-17A and Cognitive Dysfunction After Ischemic Stroke

Dementia occurs in about one-third of patients after a stroke. The incidence of vascular cognitive impairment (VCI) that does not meet the diagnostic criteria for dementia is higher. It often progresses to a dementia state without early detection and treatment, which significantly impacts patients’ lives. Studies have shown that Th17 cells and IL-17A correlate with VCI after stroke and that the proportion of Th17 and IL-17 levels in IS patients at admission are positively associated with cognitive decline 1 and 2 years after IS [24]. The authors suggested that it may be because chronic inflammation caused by Th17 cells and IL-17A leads to long-term cognitive decline in patients after IS. Th17 cells on day 3 and day 7 after onset were negatively correlated with MMSE scores at discharge [22]. In contrast, a study by Peng et al. suggested that peripheral blood IL-17 + Th17 cells in patients with stroke-induced VCI did not differ from those in the healthy population [92]. Zhang et al. statistically found that peripheral cytokines, including IL-17A, had little predictive value for the recovery of cognitive function during subacute inpatient rehabilitation after stroke [93]. The mechanism by which IL-17A and Th17 cells contribute to cognitive dysfunction may be related to the inhibition of hippocampal neuronal proliferation and neurogenesis. This has been described in detail in the previous section and will be added a little in the following. A link was found between intestinal responses and increased peripheral IL-17, especially in the context of a high-salt diet. A high salt diet promoted Th17 polarization by activating the p38/MAPK pathway and led to increased plasma levels of IL-17. IL-17 had toxic vascular effects, causing brain endothelial cells to stop producing eNOS, leading to reduced cerebral blood flow and cerebrovascular dysfunction, and ultimately to neuronal dysfunction and cognitive impairment [94]. Intestinal epithelial stem cell (Lgr5 + stem cells) transplantation reduced circulating levels of LPS and IL-17A and improved cognitive function 4 weeks after stroke by repairing the intestinal structure and decreasing intestinal permeability [95]. A more nuanced view was presented by Ribeiro, whose study showed that IL-17 KO mice exhibited short-term memory impairment while long-term memory did not show abnormalities. They believe that this may be related to IL-17A stimulating glial cells to produce BDNF and increasing hippocampal neuronal plasticity [96].

## Targeting Th17 Cells and IL-17A in Treatment of Ischemic Stroke

There are fewer studies on IL-17A and Th17 cells for treating IS. The following section focuses on an overview of the mechanisms involved in inhibiting the differentiation of Th17 cells and inhibiting/neutralizing the production of IL-17A to find therapeutic targets for IS (Table 1).

**Table 1:** Therapeutic strategies for the treatment of ischemic stroke targeting Th17 cells and IL-17A

Treatment	Method	Study type	Rodent model/cell type	Stroke model	Target/mechanism	Time	Effects	Published year	Reference
Lentiviral vectors (LVs) encoding IL-23p19 short hairpin RNA	Intravenous injection	In vivo	C57BL/6 mice	pMCAO	Knockdown of IL-23p19	5 days after ischemia	Neurological dysfunction ↓ Cerebral infarct area↓ IL-17↓ IFN-γ,Foxp3↑	2015	[97]
-	-	In vivo	IL-23R KO Mice	tMCAO	Knockout of IL-23R	3 days after tMCAO	Cerebral infarct area↓ Neutrophil infiltration↓ IL-17-producing γδ T cells↓	2018	[98]
Anti-p40 antibody	Intraperitoneal injection	In vivo	C57BL/6 mice	tMCAO	Blocks both IL-12 and IL-23	3 days after tMCAO	Neurological dysfunction ↓ Cerebral infarct area↓ IL-17-positive γδ T cells↓	2010	[99]
CP-690550	Intraperitoneal injection	In vivo	C57BL/6 mice	tMCAO	Inhibitor of JAK-3	7 days after tMCAO	Neurological dysfunction ↓ Cerebral infarct area↓	2010	[99]
Recombinant mouse IL-33 solution	Intracerebroventricular injection	In vivo	C57BL/6 mice	tMCAO	Increase IL-33	24 h and 72 h after tMCAO	Cerebral infarct area↓ IFN-γ,IL-17↓ IL-4↑	2017	[28]
1,25-VitD3	Intravenous injection	In vivo	C57BL/6 mice	tMCAO	Th17/Tregs	24 h after tMCAO	Cerebral infarct area↓ Rorc mRNA↓ IL-1β, IL-6,IL-23α,TGF-β1,Gp91phox (NOX-2) ↓ Neutrophil infiltration↓	2018	[100]
Specific miR-155 inhibitor	Intravenous injection	In vivo	C57BL/6 mice	pMCAO	Increase SOCS-1 and SHIP-1	7 days after tMCAO	p-STAT3↓ CCL12,CXCL3↓ IL-4,IL-5,IL-6,IL-10,IL-17↑	2016	[101]
						14 days after tMCAO	p-STAT3↑ CCL12,CXCL3↓ IL-4,IL-5,IL-6,IL-10,IL-17↑		
PR-957	Intragastrically	In vivo	C57BL/6 mice	tMCAO	Blockade of the LMP7	72 h after tMCAO	Neurological dysfunction ↓ Cerebral infarct area↓ p-STAT3↓ RORγt mRNA↓ CD4 + T cells, Th17 cells↓ IL-1β,IL-6,IL-12,IL-17A,TNF-α↓	2018	[29]
Anti-IL-23	Intravenous injection	In vivo	Sprague–Dawley rats	tMCAO	IL-23	24 h after tMCAO	Neurological dysfunction ↓ Cerebral infarct area↓ MDA↓ SOD,GSH-Px↑ p-JAK2,p-JAK3,p-STAT3↑	2021	[102]
Anti-IL-17A	Intravenous injection	In vivo	C57BL/6 mice	tMCAO	IL-17A	72 h after tMCAO	Cerebral infarct area↓ Neutrophil infiltration↓	2023	[103]
Fecal microbiota transplantation (FMT) from young mice	Intragastrically	In vivo	C57BL/6 mice	tMCAO	Microbiota environment	24 h after tMCAO	Neurological dysfunction ↓ Cerebral infarct area↓ IL-17↓	2021	[104]
indole-3-propionic acid	Intragastrically	In vivo	C57BL/6 mice	tMCAO	Gut metabolites	5 days after tMCAO	Neurological dysfunction ↓ Cerebral infarct area↓ Intestinal barrier function and epithelial integrity↑ Th17 cells in the gut-associated lymphoid tissue↓ IL-1β, IL-6 and CCL2↓	2022	[105]
fecal microbiota transplantation (FMT) from young mice	Intragastrically	In vivo	C57BL/6 mice	tMCAO	Microbiota environment	14 days after tMCAO	Neurological dysfunction ↓ Cerebral infarct area↓ IL-17↓	2021	[106]
Periodontitis salivary microbiota	Intragastrically	In vivo	C57BL/6 mice	tMCAO	-	24 h after tMCAO	Cerebral infarct area↑ Neutrophil infiltration↑ Th17 cells,IL-17,IL-1β↑	2022	[107]
Resveratrol	Intraperitoneal injection	In vivo	C57BL/6 mice	tMCAO	Microbiota environment	3 days after tMCAO	Neurological dysfunction ↓ Cerebral infarct area↓ Small Intestinal Epithelial and Vascular Permeability ↓ IL-17A, IFN-γ, TNF-α ↓	2019	[108]
Salidroside	Intraperitoneal injection	In vivo	Sprague–Dawley rats	tMCAO	STAT-3	24 h after tMCAO	Neurological dysfunction ↓ Cerebral infarct area↓ IL-6,TNF-α,MCP-1↓ ROR-γt, Foxp3 ↓ Th17 cells↓	2021	[109]
Dihuang Yinzi	Intragastrically	In vivo	Sprague–Dawley rats	tMCAO	Microbiota environment	7 days after tMCAO	IL-6,TNF-α,IL-17↓	2022	[110]
Glycyrrhizin	Intraperitoneal injection	In vivo	C57BL/6 mice	tMCAO	HMGB1/TLR4/IL-17A signaling pathway	72 h after tMCAO	Neurological dysfunction ↓ Cerebral infarct area↓ Neuronal apoptosis↓ IL-17A↓	2014	[111]
Xueshuantong for Injection	Intravenous injection	In vivo	Wistar rats	tMCAO	Prx6-TLR4 pathway	72 h after tMCAO	Neurological dysfunction ↓ Cerebral infarct area↓ IL-1β,IL-17,IL-23p19,TNF-α,iNOS↓	2015	[112]
Astragalus membranaceus extract and ligustrazine	Intraperitoneal injection	In vivo	Wistar rats	tMCAO	-	24 h after tMCAO	Neurological dysfunction ↓ Cerebral infarct area↓ IL-1β,IL-17, IFN-γ↓ Foxp3,TGF-β1,IL-10,IL-4↑	2019	[113]
Astragaloside IV	Intravenous injection	In vivo	C57BL/6 mice	pMCAO	Wnt pathway	7 days after pMCAO	Cognitive deficits and synapse repair↑ DCX, BrdU,Sox2↑ IL-17↓	2020	[79]
		In vitro	Primary hippocampal NSCs	-	Wnt pathway	3 days after treatment	Neurospheres proliferation↑		

### Inhibition of Proliferation and Differentiation of Th17 Cells

The proliferation and differentiation of Th17 cells are regulated by extracellular signaling, transcription, RNA modification, and post-translational modifications.

#### Altering the Microenvironment Affecting Th17 Cell Differentiation

Many studies have shown that the differentiation of Th17 cells is influenced by the extracellular microenvironment, which depends mainly on various cytokines (IL-6, IL-21, and IL-23, etc.) and immune cells (Dendritic cells). Therefore blocking the signaling of these cytokines (e.g., neutralizing antibodies, inhibitors) can inhibit Th17 differentiation and alleviate IS. When cerebral ischemia occurs, infiltrating macrophages and dendritic cells(DCs) are the main source of IL-23. In the I/R mouse model, deletion of the IL-23 gene has a more pronounced protective effect than deletion of γδ T cells [114], suggesting that IL-23 not only induces proliferation of γδ T cells after stroke but also is greatly likely to be involved in the proliferation and differentiation of Th17 cells. Lentiviral shRNA specially targeting IL-23p19 effectively inhibits the IL-23/IL-17 axis, reduces IL-17 expression in brain tissue 5 days after pMCAO, and ultimately improves neurological scores and reduces infarct volume [97]. Depletion of IL-23-producing CD172a + /Irf4-Expressing cDC2 Cells similarly ameliorates neurological deficits after stroke [98]. Monoclonal antibodies against IL-12 and IL-23 P40 reduce CD4 + T cells and γδ T cells in the brains of I/R mice and attenuate infarct volume and neurological deficits [99]. Luo et al. showed that IL-33 decreased in the ischemic brain of mice 6 h after tMCAO. And they found that exogenous ventricular injection of IL-33 reduced the proportion of IL-17-secreting T cells in brain tissue and improved neurological function [28].

#### Reduction in the Levels of Relevant Transcription Factors

At the transcriptional and RNA levels, most of the current therapeutic targets are focused on transcription factors and miRNAs. The extracellular environmental factors mentioned above can promote IL-17 expression by activating the transcription factor RORγt through the JAK2-STAT3 signaling pathway or SMAD2. In this process, RORγt, RORc, STAT3, SMAD2, BATF, and IRF4 can directly bind to IL-17 gene promoter, promote IL-17 gene transcription, and regulate Th17 cell differentiation. Inhibition of the above transcription factors can inhibit Th17 differentiation and IL-17A secretion and improve the symptoms of neurological deficits. Megan et al. reduced the transcription factor RORc in Th17 cells and decreased infarct volume by giving tMCAO mice 1,25-VitD3, the active form of vitamin D3 [100]. MiR-155 promoted Th17 cell differentiation by targeting the inhibition of cytokine signaling 1 (SOCS1), which can inhibit JAK-STAT3 signaling. MiR-155 inhibitor significantly reduced the expression of phosphorylated STAT3 in the brains of distal MCAO (dMCAO) mice during the subacute phase (7 to 14 days) and attenuated neuroinflammation [101].

#### Inhibition of the Activity of Related Transcription Factors

The transcription factors STAT3 and RORγt, which are involved in Th17 differentiation, are regulated by various post-translational modifications. Non-phosphorylated STAT3 is not transcriptionally active and is activated only after phosphorylation by JAK2 on the cell membrane. The JAK kinase inhibitor CP-690550 inhibited IL-17 production by γδ T cells and activated CD4 + T cells, reducing infarct volume in tMCAO mice. PR-957 (also named ONX 0914), a selective inhibitor of the immunoproteasome subunit LMP7, strongly attenuated brain injury and inhibited pro-inflammatory cytokine activity [115]. Guo et al. showed that PR-957 downregulated p-STAT3 protein expression in the brain, leading to a decrease in RORγt at the transcriptional level after tMCAO and ultimately leading to the inhibition of Th17 cell differentiation and IL-17A secretion [29]. However, a study by Fan et al. showed that IL-23 antibodies enhanced the phosphorylation levels of JAK2 and STAT3 and reduced the infarct volume in tMCAO mice. The ameliorative effect of neurological deficits was abolished when JAK2 inhibitors were used in conjunction with IL-23 antibodies [102]. Post-transcriptional regulation of Th17 cell differentiation is an important target for treating stroke, and more studies are needed to elucidate it further.

### Neutralization or Inhibition of IL-17A

Many studies have shown that inhibition of IL-17A secretion or neutralization of IL-17A using monoclonal antibodies has promising therapeutic effects in treating IS and is a good therapeutic approach. Monoclonal anti-murine IL-17A antibody is widely used in treating MCAO mice and has significantly improved. Anti-IL-17A treatment significantly inhibits neutrophil infiltration, reduces the activation of microglia and astrocytes, and reduces the infarct volume in the acute phase of MCAO [59, 61, 66]. Notably, a multicenter preclinical randomized controlled trial by Gelderblom et al. showed that anti-interleukin-17A reduced infarct volume in large infarct lesions (involving both cortical and striatal regions) but had no significant effect on small infarct lesions (involving only striatal regions) [103]. Suckinumab, ixekizumab, and brodalumab are monoclonal antibodies that inhibit IL-17A and are widely used in the treatment of psoriasis and ankylosing spondylitis (AS). The restoration of neuronal cell death produced by Th17 cells in Parkinson’s patient-generated pluripotent stem cell-derived neurons by secukinumab [116]. Unfortunately, there are no studies on the use of the above drugs for the treatment of IS.

A large body of evidence suggests that intestinal flora is essential for normal host metabolism and physiological function, affecting the immune and nervous systems. The gut microbiota affects the gut-brain axis via immune (cytokines), endocrine (cortisol), and neural (enteric nervous system) pathways [117]. Lvanov et al. demonstrated the importance of intestinal flora, especially segmented filamentous bacteria (SFB), in mucosal Th17 cell production [118]. In recent years, there have been an increasing number of studies related to the effects of microorganisms and their metabolites on IS through modulation of the cytokine IL-17A. After 30 days of continuous gavage of fecal flora from young mice to aged mice, researchers established the tMCAO model. They found significant reductions in IL-17 levels in serum, colon, and brain tissue, reduced infarct volumes, and improved neurological function [104]. Indole-3-propionic acid (IPA), a tryptophan (Trp) catabolic product produced by intestinal flora, is reduced in the serum of MCAO mice. Significant increases in the number of Th17 and significant decreases in the number of Treg in tMCAO-induced Peyer’s patches and intestine-associated lymphoid tissues were reversed by exogenous supplementation of IPA [105]. Short-chain fatty acids (SCFAs) are produced by bacteria when they metabolize non-digestible fibers in the intestine and act to stabilize the intestine by activating G protein-coupled receptors and inhibiting histone deacetylases [119]. SCFAs can ameliorate neurological damage by enhancing the integrity of the intestinal barrier and attenuating the inflammatory response in the gut and brain. The mechanism is related to changes in the expression profile of various cytokines necessary to mediate the inflammatory response and immune cell maturation, including inhibition of pro-inflammatory cytokines: IL-17, TNF-α, MCP-1, and IL-1β [120]. Lee et al. showed that restoring SCFA to levels found in young microbiomes using SCFA-producing bacteria resulted in increased brain and plasma SCFA levels, decreased brain IL-17 + γδ T cells and IL-17A, and significantly improved stroke outcomes [106]. Microflora from the oral cavity spreads to the intestine with swallowing. Chen’s study showed that MCAO model mice gavaged with salivary flora of periodontitis exhibited more severe neuroinflammation and worse prognosis. The mechanism may be related to increased IL-17A-producing immune cells (including Th17 cells and IL-17 + γδ T cells) in the gut and facilitated the migration of these cells from the gut to the brain [107].

### Traditional Chinese Medicine Treatment Targeting Th17 Cells or IL-17A

Traditional Chinese medicine (TCM) has a long history of the treatment of stroke in China with remarkable efficacy. Chinese herbal medicine, including formulas, extracts, and compounds, is characterized by integrated treatment with multiple sites and targets and overall regulation for the treatment of IS. IL-17A has been widely studied as an important inflammatory factor. The following section summarizes the published studies on TCM that can improve stroke by modulating Th17 cells or IL-17A.

Resveratrol, a natural polyphenol, reduces BBB injury and neuroinflammation and improves neurological deficits in tMCAO mice. Dou et al. showed that Resveratrol improved tMCAO-induced increase in small intestinal epithelial and vascular permeability, attenuated the increase in Th17 cells in the lamina propria of the small intestine, and attenuated the increase in IL-17A in serum and brain tissue by modulating intestinal flora [108]. Salidroside (Sal) decreased the expression of RORγt and the number of Th17 cells in the peripheral circulation and increased the number of peripheral Treg cells in ischemic brain tissue. In hypoxic T cells (Th17 and Treg cells), Sal significantly inhibited the expression of IL-6, TNF-α, MCP-1, STAT-3, and NF-κB proteins [109]. Dihuang Yinz can regulate the intestinal flora of tMCAO rats to the firmicutes, bacteroidetes, and proteobacteria, reduce the content of IL-6, TNF-α, and IL-17, and increase the content of TGF-β, IL-10 in the brain, serum, and colon tissues [110]. HMGB1/TLR4 signaling pathway can induce IL-17A secretion [42]. Glycyrrhizin significantly reduces infarct volume and neurological deficits at 3 days after MCAO by inhibiting the HMGB1/TLR4/IL-17A signaling pathway [111]. Xueshuantong for Injection attenuates tMCAO-induced infarct volume and edema and reduces the expression of IL-17, IL-23p19, IL-1β, and TNF-α mRNA in the brain by inhibiting the Prx6-TLR4 signaling pathway [112]. Hyperforin, a pharmacologically active component of the medicinal plant Hypericum perforatum, reduces IL-17A expression and IL-17A-mediated microglia activation after 3 days of tMCAO onset to alleviate acute cerebral ischemic injury [61]. A combination of Astragalus membranaceus extract and ligustrazine improves neuroinflammation in rats with cerebral ischemia treated with thrombolysis by increasing the expression of endogenous Tregs and decreasing inflammatory factors such as IL-17A and IL-1β [113]. In addition to the effects of IS in the acute phase, IL-17A and Th17 cells also play a role in neurogenesis and angiogenesis in the subacute and recovery phases of IS. Astragaloside IV (As IV), a primary bioactive compound of Radix Astragali, may inhibit IL-17A expression and upregulate Akt/GSK-3β and Wnt/β-catenin signaling pathway proteins by decreasing the expression of IL-17A and neuronal apoptosis and promote neurogenesis in MCAO mice, ultimately alleviating post-stroke cognitive impairment [78, 79]. Zhang et al. showed that Hyperforin promotes angiogenesis and improves prognosis in tMCAO mice at 28 days. However, they suggested that the possible mechanism was that Hyperforin induced an increase in IL-17A and further promoted vascular endothelial growth factor (VEGF) expression [86]. This study presents a contrary opinion to the studies with lowering IL-17A as a therapeutic target. Therefore the further investigation is still needed for the therapeutic mechanism of IL-17A in stroke recovery.

## Conclusion

Published studies suggest that IL-17A and Th17 cells have important effects on the pathogenesis, secondary brain injury, and regulation of the prognosis of IS. Immunotherapy targeting IL-17A and Th17 cells has shown good ameliorative effects in ischemic stroke mice. However, it must be acknowledged that the mechanisms involved are certainly complex, and many unresolved questions need to be addressed. Th17 cells and IL-17A have prominent pleiotropic properties in regulating pro- and anti-inflammatory responses after stroke. It is important to understand the pathophysiological role of IL-17A in the acute to recovery phase of ischemic stroke and to suggest appropriate therapeutic approaches. Whether other members of the IL-17 family are involved in the post-stroke pathological process still needs to be explored. Nevertheless, the available studies have shown that biological therapies targeting IL-17A may be novel therapies for the treatment of IS and deserve further investigation.

## Data Availability

The data on which the review is based were accessed from a repository and are available for downloading through the following link: PubMed (nih.gov).
